# Supplementary material for: Large-area, untethered, metamorphic, and omnidirectionally stretchable multiplexing self-powered triboelectric skins
Source: Nat Commun. 2024 Feb 9;15:1238. doi: 10.1038/s41467-024-45611-6 (PMC10858173; doi:10.1038/s41467-024-45611-6)
Supplement: Supplementary file 1 — Supplementary Information [file 41467_2024_45611_MOESM1_ESM.pdf]

Supporting Information for

Large-Area, Untethered, Metamorphic, and Omnidirectionally-Stretchable Multiplexing Self-Powered Triboelectric Skins

Beibei Shao[1,2#], Ming-Han Lu[3#], Tai-Chen Wu[3], Wei-Chen Peng[3], Tien-Yu Ko[3], Yung-Chi Hsiao[3], Jiann-Yeu Chen[4], Baoquan Sun[1,2,5], Ruiyuan Liu[1,2]*, Ying-Chih Lai[3,4,6]**

[1]Soochow Institute of Energy and Material Innovations, Key Laboratory for Advanced Carbon Materials and Wearable Energy Technologies of Jiangsu Province, Institute of Functional Nano & Soft Materials (FUNSOM) and College of Energy, Soochow University, Suzhou 215006, P. R. China.

[2]Jiangsu Key Laboratory of Advanced Negative Carbon Technologies, Soochow University, Suzhou 215123, P. R. China.

[3]Department of Materials Science and Engineering, National Chung Hsing University, Taichung, 40227, Taiwan

[4]Innovation and Development Center of Sustainable Agriculture, i-Center for Advanced Science and Technology, National Chung Hsing University, Taichung, 40227, Taiwan

[5]Macau Institute of Materials Science and Engineering MUST-SUDA Joint Research Center for Advanced Functional Materials Macau University of Science and Technology Macau 999078, P. R. China.

[6]Department of Physics, National Chung Hsing University, Taichung, 40227, Taiwan

This Supporting Information file includes the following sections:

Supplementary Tables S1

Supplementary Figures S1 to S15

Supporting Information 1. Performance summary of TENG-based structures

Table S1. Comparisons of UTE-skin with state-of-the-art TENG-based structures

Year	Device structures	Misrecognition rate/%	Recognition rate/%	Flexibility	Stretchability	Reference
2016	PDMS/electrode/EVA/top PET/bottom PET	40.0	60.0	√	×	Ref.[1]
2018	shield layer/PDMS/PMMA-PDMS/Carbon black-PDMS	12.5	87.5	√	×	Ref.[2]
2018	shield layer/PET/ITO/silicone	25.0	75.0	√	uniaxial stretchability	Ref.[3]
2020	tribolayer/electrode/shield layer/insulating layer/spacer	16.0	84.0	√	×	Ref.[4]
2020	Al/Ecoflex/PVA/PEI	0.30	99.7	√	×	Ref.[5]
2021	shield layer/PDMS/PI/Cu/PI/PDMS	5.60	94.4	√	30% uniaxial stretchability	Ref.[6]
2023	Ecoflex/shield layer/Ecoflex-carbon blacks/Ecoflex	0.20	99.8	√	100% uniaxial, 100% biaxial, and 400% isotropic stretchability	**This work**

Supporting Information 2. Mechanical testing of UTE-skin and corresponding functional layers

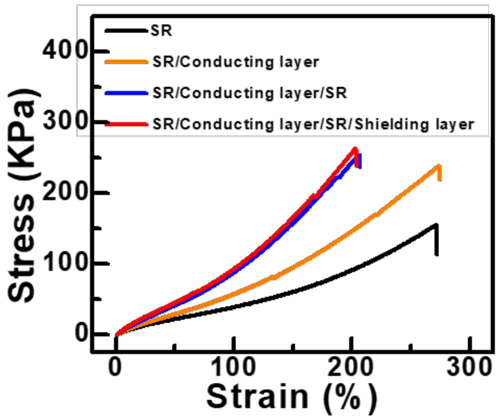

Fig. S1. Stress-strain curves of the Ecoflex silicone rubber (SR), SR/conducting layer, SR/conducting layer/SR, and SR/conducting layer/SR/shielding layer, respectively.

Supporting Information 3. Mechanical testing of the UTE-skin during 100 times stretching

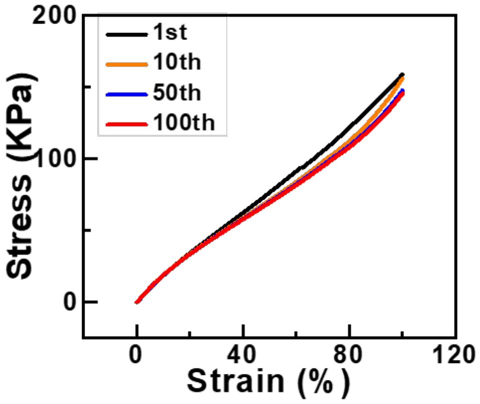

Fig. S2. Stress-strain curves of the UTE-skin during 100 times repeated tests.

Supporting Information 4. Electrical characterizations of misrecognition suppression by the shielding layer depending on its thickness

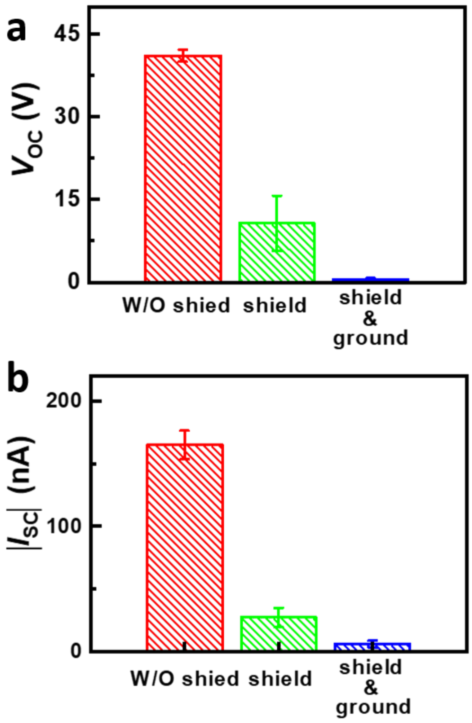

Fig. S3. Comparison of the output (a) voltage and (b) current of the sensing nodes and elastic circuits with shielding layers (without ground and with ground), respectively. Error bars in (a and b) represent standard deviations, n = 3 independent samples.

Supporting Information 5. Electrical characterizations of misrecognition suppression by the shielding layer depending on its thickness

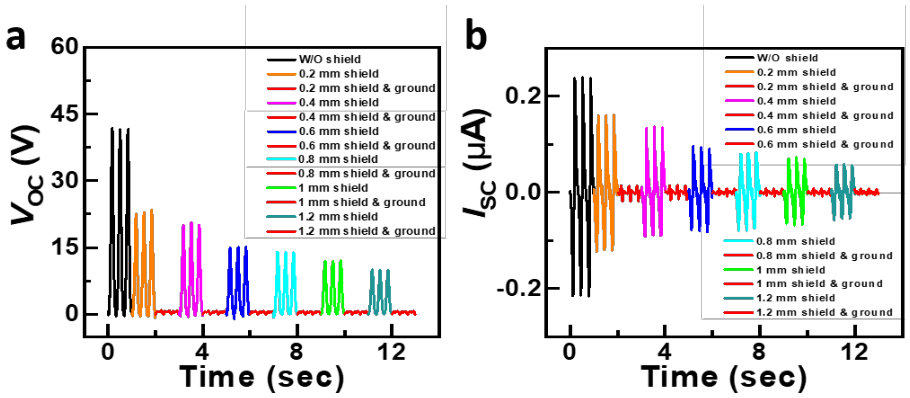

Fig. S4. Comparison of the output (a) voltage and (b) current of the elastic circuits with shielding layers upon different thicknesses, respectively.

Supporting Information 6. Electrical performance characterization under uniaxial, biaxial, and isotropic tensile strains

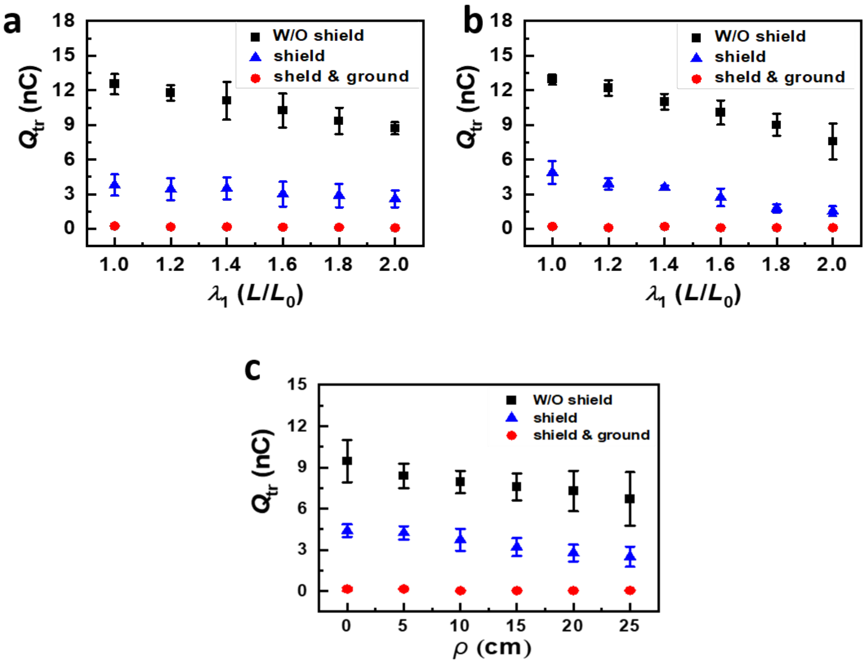

Fig. S5. Transferred charges (Q_{tr}) of the sensing node as a function of (a) uniaxial strain, (b) biaxial strain, and (c) isotropic strain. Error bars in (a, b and c) represent standard deviations, $n = 3$ independent samples.

Supporting Information 7. Outputs dependence of the self-powered TENG on different contact time

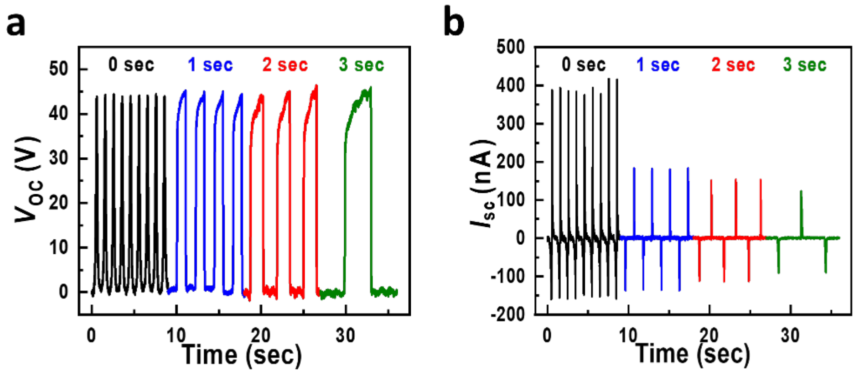

Fig. S6. Dependence of (a) V_{oc} and (b) I_{sc} of the sensing node of UTE-skin on different contact time.

Supporting Information 8. Schematic illustration of the working mechanism of UTE-skin before and after stretching

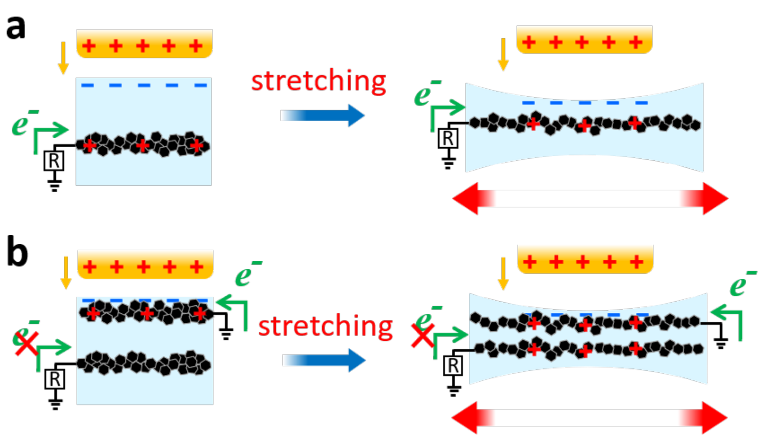

Fig. S7. Schematic illustration of the working mechanism of the UTE-skin without (a) and with (b) a top shielding layer after stretching, illustrating that the conductivity of the carbon-blacks-doped shielding layer correlates with the degree of carbon particle percolation.

Supporting Information 9. Output voltage of the stretchable self-powered TENG during 1000 cycles of repeated stretching

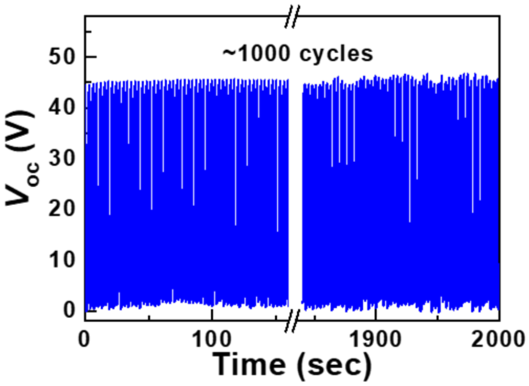

Fig. S8. Output voltage of the stretchable self-powered TENG during 1000 cycles of repeated stretching, illustrating its mechanical reliability.

Supporting Information 10. Resistance of the shielding layer during 1000 cycles of repeated stretching

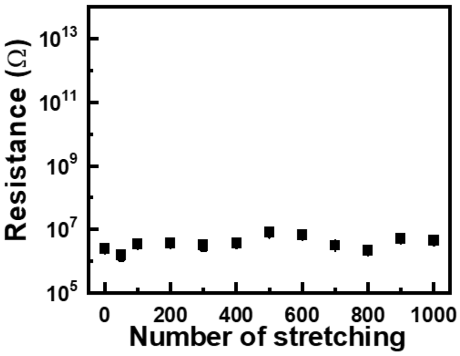

Fig. S9. Resistance of the shielding layer during 1000 cycles of repeated stretching.

Supporting Information 11. Outputs dependence of the self-powered TENG on different kinds of contact materials

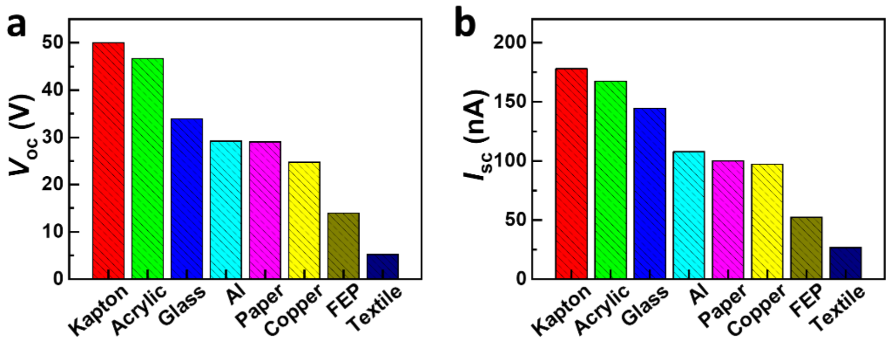

Fig. S10. Dependence of (a) output voltage and (b) current of the sensing node of UTE-skin on different kinds of contact materials.

Supporting Information 12. Outputs dependence of the self-powered TENG on different operating frequencies

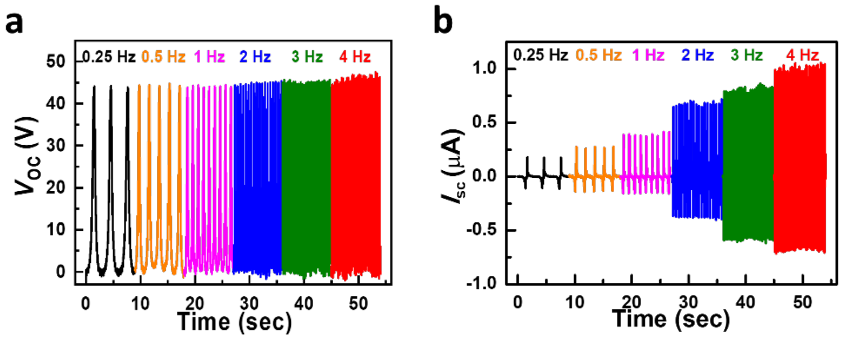

Fig. S11. Dependence of (a) output voltage and (b) current of the sensing node of UTE-skin on different operating frequencies.

Supporting Information 13. Outputs dependence of the self-powered TENG on different applied pressures

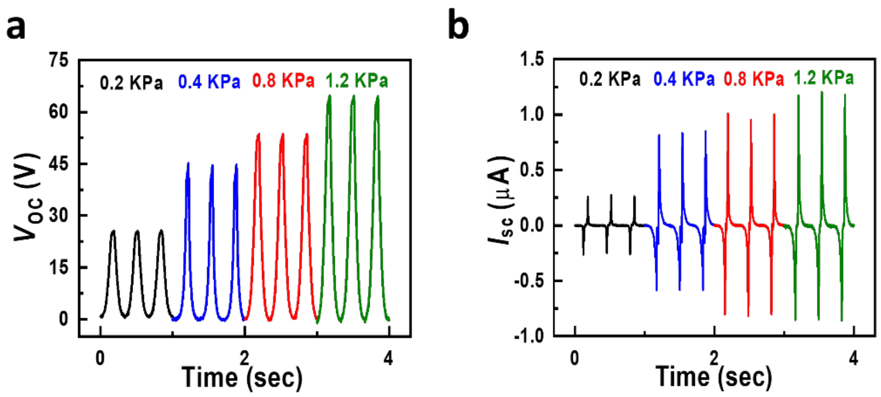

Fig. S12. Dependence of (a) output voltage and (b) current of the sensing node of UTE-skin on different applied pressures.

Supporting Information 14. Outputs dependence of the self-powered TENG on different applied pressures

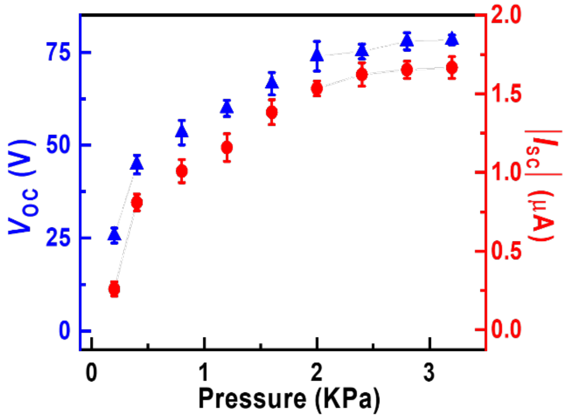

Fig. S13. Pressure-dependent output voltage and current of the sensing node of UTE-skin.

Supporting Information 15. Applications of TENG as an energy harvester

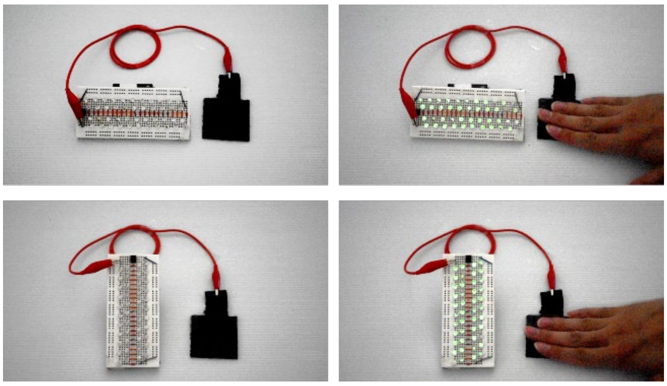

Fig. S14. Photograph showing that 40 commercial green light emitting diodes (LEDs) were lit up when the device was touched.

Supporting Information 16. Applications of TENG as an energy harvester

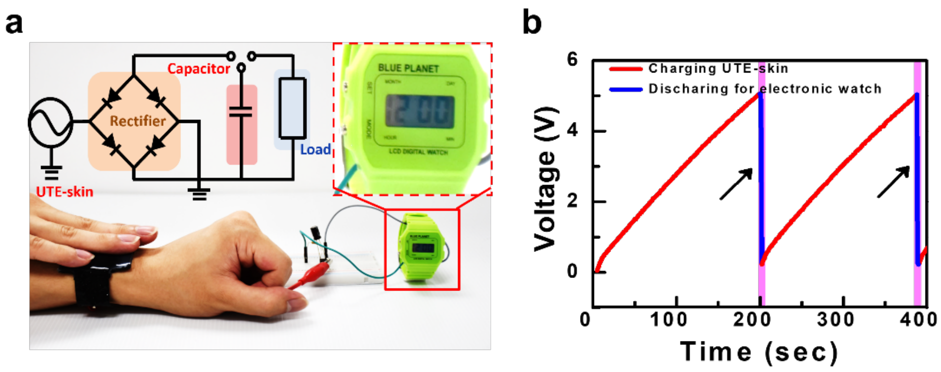

Fig. S15. Demonstration of (a) collecting mechanical energy from touching to power an electronic watch and (b) corresponding real-time charge/discharge curve.

References

1. Wang, X.*, et al.* Self-powered high-resolution and pressure-sensitive triboelectric sensor matrix for real-time tactile mapping. *Adv. Mater.* **28**, 2896 (2016).
2. Ren, Z.*, et al.* Fully Elastic and Metal-Free Tactile Sensors for Detecting both Normal and Tangential Forces Based on Triboelectric Nanogenerators. *Adv. Funct. Mater.* **28**, 1802989 (2018).
3. Wu, C.*, et al.* Keystroke dynamics enabled authentication and identification using triboelectric nanogenerator array. *Mater. Today* **21**, 216 (2018).
4. Pu, X.*, et al.* Flexible triboelectric 3D touch pad with unit subdivision structure for effective XY positioning and pressure sensing. *Nano Energy* **76**, 105047 (2020).
5. Wang, L.*, et al.* A metal-electrode-free, fully integrated, soft triboelectric sensor array for self-powered tactile sensing. *Microsyst. Nanoeng.* **6**, 59 (2020).
6. He, J.*, et al.* Trampoline inspired stretchable triboelectric nanogenerators as tactile sensors for epidermal electronics. *Nano Energy* **81**, 105590 (2021).
