# Supplementary material for: Large-area, untethered, metamorphic, and omnidirectionally stretchable multiplexing self-powered triboelectric skins
Source: Nat Commun. 2024 Feb 9;15:1238. doi: 10.1038/s41467-024-45611-6 (PMC10858173; doi:10.1038/s41467-024-45611-6)
Supplement: Supplementary file 3 — Description of Additional Supplementary Files [file 41467_2024_45611_MOESM3_ESM.pdf]

Description of Additional Supplementary Files

Supplementary Movie Legends

File Name: Supplementary Movie S1
Description: Demonstration of UTE-skin as a power supply for actuating LED arrays

File Name: Supplementary Movie S2
Description: System-level demonstration of UTE-skin as a self-powered human-system interface for controlling a music player

File Name: Supplementary Movie S3
Description: System-level demonstration of UTE-skin as a self-powered human-system interface for playing a video game

File Name: Supplementary Movie S4
Description: System-level demonstration of UTE-skin as a self-powered human-system interface for controlling a wearable smartphone keypad
